# Coordinated increase of γ-secretase reaction products in the plasma of some female Japanese sporadic Alzheimer's disease patients: quantitative analysis of p3-Alcα with a new ELISA system

**DOI:** 10.1186/1750-1326-6-76

**Published:** 2011-11-08

**Authors:** Tomoko Konno, Saori Hata, Yukiko Hamada, Yuko Horikoshi-Sakuraba, Tadashi Nakaya, Yuhki Saito, Tohru Yamamoto, Takayuki Yamamoto, Masahiro Maeda, Takeshi Ikeuchi, Sam Gandy, Hiroyasu Akatsu, Toshiharu Suzuki

**Affiliations:** 1 Laboratory of Neuroscience, Graduate School of Pharmaceutical Sciences, Hokkaido University, Sapporo 060-0812, Japan; 2 Immuno-Biological Laboratories Co., Ltd. (IBL), Fujioka 375-0005, Japan; 3 Choju Medical Institute, Fukushimura Hospital, Toyohashi 441-8124, Japan; 4 Department of Molecular Neuroscience, Brain Research Institute, Niigata University, Niigata 951-8585, Japan; 5 Department of Neurology, Mount Sinai School of Medicine, Alzheimer's Disease Research Center, New York, NY 10029, USA; 6 Department of Psychiatry, Mount Sinai School of Medicine, Alzheimer's Disease Research Center, New York, NY 10029, USA; 7 James J. Peters Veterans Administration Medical Center, NY 10468, USA

## Abstract

**Background:**

Aggregatable amyloid β-peptide (Aβ) and non-aggregatable p3-Alcα are metabolic products of the γ-secretase cleavage of amyloid β-protein precursor (APP) and Alcadeinα (Alcα), respectively. Familial AD (FAD) -linked mutations in the presenilin 1 or 2 (PS1 or PS2) component of γ-secretase can cause alternative intramembranous processing of APP and Alcα, leading to a coordinated generation of variants of both Aβ and p3-Alcα. Variant Alcα peptides have been observed in the cerebrospinal fluid (CSF) of patients with mild cognitive impairment and sporadic Alzheimer's disease (AD). Since, like APP, Alcα is largely expressed in brain, one might predict that alternative processing of Alcα would be reflected in body fluids of some AD patients. These patients with misprocessing of multiple γ-secretase substrates might define an endophenotype of p3-Alcα, in whom AD is due either to dysfunction of γ-secretase or to a disorder of the clearance of hydrophobic peptides such as those derived from transmembrane domains.

**Results:**

We developed a simple procedure for extraction of p3-Alcα from plasma and for analyzing this extract in a sensitive, p3-Alcα-specific sandwich enzyme-linked immunosorbent assay (ELISA) system. Plasma p3-Alcα levels and Aβ40 levels were examined in sporadic AD subjects from two independent Japanese cohorts. In some of these patients, levels of plasma p3-Alcα were significantly higher, and were accompanied by parallel changes in Aβ40 levels. This AD-related difference was more marked in female subjects, but this phenomenon was not observed in subjects with frontotemporal lobar degeneration (FTLD).

**Conclusion:**

Reagents and procedures have been established that enable extraction of p3-Alcα from plasma and for quantification of plasma p3-Alcα levels by ELISA. Some populations of AD subjects apparently show increased levels of both p3-Alcα and Aβ40. Quantification of p3-Alcα level may be useful as a readily accessible biomarker for a population of sporadic AD patients in which disease pathogenesis is associated with either dysfunction of γ-secretase or with a disorder of the clearance of transmembrane domain-derived peptides.

## Background

Alcadein proteins (Alcs) represent a family of neuronal type I transmembrane proteins composed of Alcadein_α _(Alc_α_), Alcadein_β _(Alc_β_) and Alcadein_γ _(Alc_γ_). The Alcs are encoded by three independent genes that are highly conserved among mammals, as is the gene encoding the Alzheimer's amyloid β-protein precursor (APP). Alcs and APP are largely co-localized in brain neurons where they are also frequently cross-linked by the X11L adaptor. Both Alc and APP accumulate in the dystrophic neurites surrounding senile plaques of Alzheimer's disease (AD) brain and participate in the molecular pathobiology of the disease [1-5]. Although Alcs undergo alternative processing in sporadic AD [5], our data indicate that Alcs are not aggregatable (unpublished observations), and Alcs do not appear in amyloid deposits [1].

Both APP and the Alcs are substrates for primary proteolytic cleavage by the APP α-secretases ADAM10 and ADAM17. These cleavages are directed toward the holoproteins within their respective intralumenal juxtamembrane domains, causing shedding of the ectodomains and retention of membrane-bound carboxyl terminal fragments (CTFs) [4]. Next, the identical γ-secretase can cleave either the APP-CTF or the Alc-CTF within the transmembrane region. This sequential α- and γ-cleavage of APP yields the p3 peptide, and, by analogy, the α- and γ-cleavage of Alc generates the p3-Alc peptide (amino acid sequence of p3-Alc_α _along with Aβ and p3 are shown in Additional file 1, **Figure S1A**).

A substantial body of research has established that pathogenic mutations in the presenilin 1 (PS1) and presenilin 2 (PS2) genes underlie most familial Alzhemer's disease (FAD: [6]), and that PS1 or PS2 forms the catalytic site of the γ-secretase aspartyl proteinase complex [7]. There are ~200 known pathogenic PS1/PS2 mutations, and all are believed to act by shifting registration of the intramembranous cleavage of APP within the catalytic site of γ-secretase so as to increase the ratio of the level of the minor and more aggregation prone Aβ species (Aβ42) relative to that of the major and less aggregatable species (Aβ40) (also known as the Aβ42/40 ratio; [8,9]). While there is controversy around whether PS mutations act by elevating Aβ42, by decreasing Aβ40, or both, there is consensus around the concept that increases in the Aβ42/40 ratio are believed to lead to AD by permitting the formation of neurotoxic Aβ oligomer(s) [10,11].

Recently, low cerebrospinal fluid (CSF) Aβ42 [12,13] and low plasma Aβ42/40 [14-16] have been identified as possible biomarkers for AD. Aβ42 levels are determined by a composite of Aβ42 generation, aggregation, and deposition. In order to study the role of γ-secretase catalysis apart from the effects of Aβ42 aggregation, we [4,5] and others [17] have studied metabolism of non-APP-derived, nonamyloidogenic γ-secretase reaction products. Using a cell culture model, we have demonstrated that Alc_α _cleavage by γ-secretase is modulated by a panel of FAD-linked PS1 mutations, all of which increased the proportionate representation of minor p3-Alc_α _species with variant C-termini [4]. Thus, we have proposed that levels and/or ratios of p3-Alc_α _species in CSF may have clinical utility as surrogate markers of γ-secretase dysfunction [5]. Conceivably, patients with AD due to γ-secretase dysfunction might be most likely to respond to γ-secretase modulator therapies.

Reduction in clearance of CSF Aβ has recently been associated with sporadic AD (SAD; [18]), a phenomenon that might well be explained by Aβ aggregation or oligomerization, since there is clear evidence that aggregated Aβ is cleared more slowly than non-aggregated forms. A less likely, but not impossible, scenario that could lead to elevated levels of Aβ and p3-Alc species involves a formulation wherein SAD might, in some cases, be attributable to a defect in clearance of transmembrane (TM) domain-derived peptides. This formulation would dovetail well with evidence that apolipoprotein E (*APOE*) isotype modulates Aβ clearance [19,20] and raises the possibility that *APOE *genotype may modulate clearance of many TM domain-derived peptides, including Aβ and p3-Alc. In separate work still in progress, we are studying whether there exists any relationship between *APOE *genotype in the levels of plasma and CSF p3-Alc. In any event, there is substantial support for the possibility that alterations in Aβ generation and/or clearance are considered to be possible underpinnings for at least some cases of SAD. These alternative hypotheses are not mutually exclusive and could underlie different biochemical endophenotypes that all lead to clinical AD.

Here we report the establishment of the reagents and procedures that will enable the extraction of p3-Alc_α _from plasma and the quantification of plasma p3-Alc_α _levels by ELISA. We also investigate the possibility that quantification of plasma levels of both p3-Alc_α _and Aβ40 may be useful as readily accessible biomarkers for defining endophenotypes of sporadic AD that are associated with either a dysfunction of γ-secretase or with a disorder of the clearance of TM domain-derived peptides.

## Results

### Extraction and quantification of serum and plasma p3-Alcs

Supplementary Figure S1A displays the amino acid sequences and cleavage sites of human p3-Alc_α _and Aβ. Non-aggregatable p3-Alc peptides are detectable in CSF [4], and changes in their quality and quantity have been proposed to represent evidence for γ-secretase dysfunction in SAD ([5]; **Hata *et al ***submitted for publication). Because circulating biomarkers offer advantages over CSF biomarkers for noninvasive and/or serial sampling, we sought to detect p3-Alc_α _in serum and plasma.

In a pilot study, efforts focused on detecting endogenous p3-Alc_α _in serum. Healthy human serum (HS), newborn calf serum (CS), fetal bovine serum (FBS) and bovine serum albumin solution (BSA, 10 mg/mL in PBS), or these same preparations containing synthetic p3-Alc_α_35 (10 ng) were subjected to immunoprecipitation after samples were depleted of immunoglobulins by pre-clearing with protein-G Sepharose beads (GE Healthcare). The immunoprecipitates were probed by Western blotting. Endogenous p3-Alc_α _was detected in FBS, while it was not detected in HS, CS, or the control BSA solution. These data indicate that, at least sometimes, endogenous p3-Alc_α _is detectable in serum (Additional file 1, **Figure S1B**).

Although endogenous p3-Alc_α _was detectable in serum, this fluid contained factor(s) that interfered with the immunodetection of p3-Alc_α _(Additional file 1, **Figure S1B**). When serum spiked with synthetic p3-Alc_α_35 peptide (10 ng) was analyzed, a strong signal was detected in the FBS and BSA solutions. However, p3-Alc_α_35 peptide was not initially detectable in HS or CS even after spiking with synthetic p3-Alc_α_35 peptide, although overexposure of the film revealed a weak signal in HS (Additional file 1, **Figure S1B**). Therefore, this pilot study suggested that; (i) endogenous p3-Alc is present in serum, but that (ii) serum contains factor(s) other than IgG that interfere with one or more steps in the immunoprecipitation/immunodetection protocol for detecting either endogenous or exogenous p3-Alc_α_.

**Figure 1:**
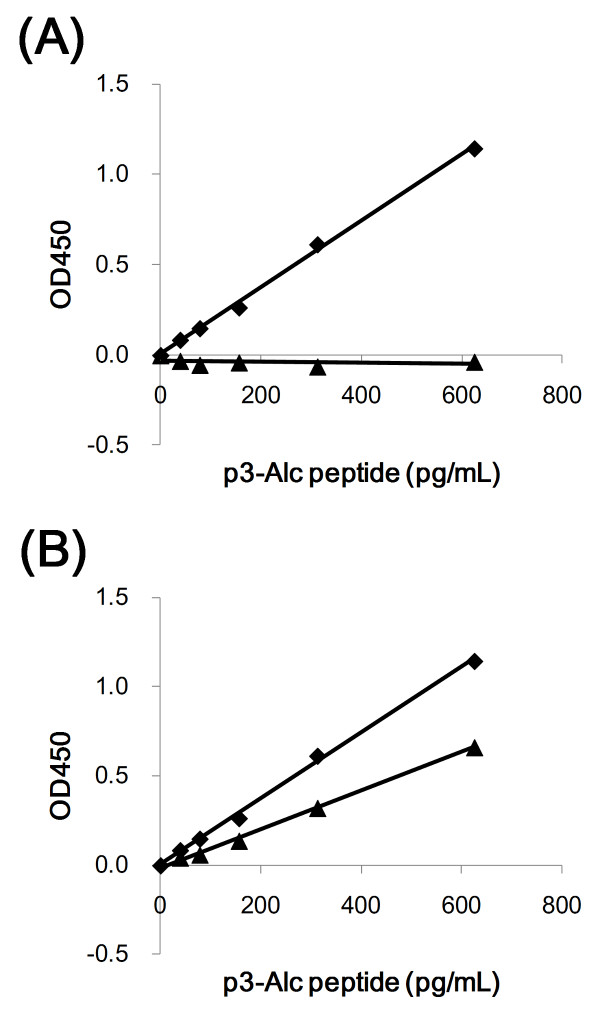
**Specificity of sELISA**. **A**. The indicated amounts (39 to 625 pg/ml) of synthetic p3-Alc_α_35 (diamonds) and p3-Alc_β_37 (triangles) peptides were dissolved in buffer A (100 μL per well of a 96-well plate) and assayed with ELISA. **B**. The indicated amounts (39 to 625 pg/ml) of synthetic p3-Alc_α_35 (diamonds) and p3-Alc_α_39 (triangles) peptides were dissolved in buffer A (100 μL per well of a 96-well plate) and assayed with ELISA and cross-reactivity was examined. The amino acid sequence information of p3-Alc_α_35 (Additional file 1, Figure S1A) and p3-Alc_β_37 are described previously (**4, 5**).

Next, we developed an ELISA system that was specific for p3-Alc_α_. We established that this ELISA could detect synthetic p3-Alc_α_35 in aqueous buffer in the range of 39 to 625 pg/mL and did not cross-react with p3-Alc_β_37, a p3-Alc_β _species derived from Alc_β _(Figure 1A). However, this ELISA was not specific for p3-Alc_α_35 since the assay also detected the p3-Alc_α _species p3-Alc_α_39 (Figure 1B). Therefore, we concluded that the ELISA could reliably be used to quantify the major total plasma p3-Alc_α _but the primary antibody was not p3-Alc_α _variant-specific.

**Figure 2:**
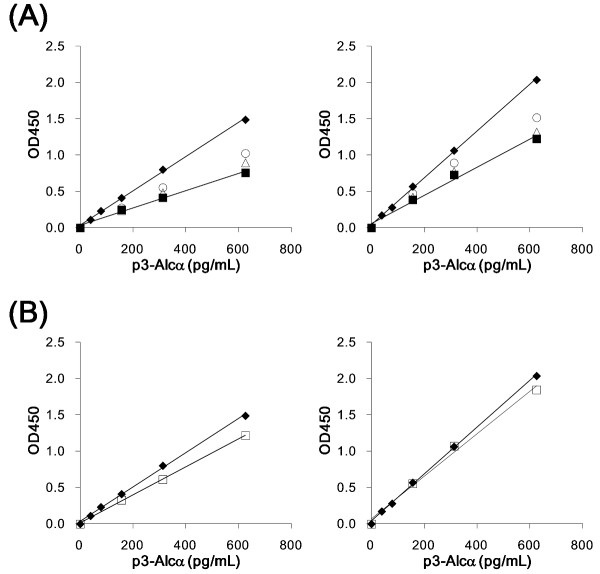
**Effect of p3-Alc_α _extraction from serum and plasma on the quantification of p3-Alc_α _with sELISA**. **A**. Quantification of p3-Alc_α_35 peptide in human serum (left) and plasma (right) without extraction. Synthetic p3-Alc_α_35 peptide was dissolved in human serum and plasma and the samples in the indicated concentrations, and samples were assayed by ELISA alongside samples in which standard p3-Alc_α_35 peptide was dissolved in buffer A (closed diamonds in each respective panel). The p3-Alc_α_35 peptide was added to undiluted serum or plasma [closed squares], or to serum or plasma diluted 2-fold [open triangles] or 4-fold [open circles] with buffer A. **B**. p3-Alc_α_35 peptide was added to undiluted serum (left) or plasma (right), and extracted using the procedure described in "***Materials and Methods ***". Extracted samples (open squares in panel B) were also examined alongside a standard p3-Alc_α_35 peptide dissolved in buffer A (closed diamonds). Endogenous p3-Alc_α _levels in serum and plasma (no addition of synthetic p3-Alc_α_) were subtracted in order to determine the specific curve for quantifying levels of synthetic p3-Alc_α_.

We next examined whether synthetic p3-Alc_α_35 in human serum and plasma could be quantified by ELISA (Figure 2). p3-Alc_α_35 was dissolved in human serum (left) or plasma (right), or in buffer A (closed diamonds), and the samples were applied to ELISA plates. Although human serum and plasma were diluted two-fold (open triangles) and four-fold (open circles) with buffer A, or left neat (closed squares), immunoreactivity to p3-Alc_α_35 was significantly suppressed under both these buffer conditions (Figure 2A), as suggested by an immunoprecipitation study (Additional file 1, **Figure S1B**). The yields of p3-Alc_α_35 (present in serum at a concentration of 625 pg/mL) were 51% (for neat samples), 60% (for samples diluted two-fold) and 69% (for samples diluted four-fold). The yields from a sample of plasma containing 625 pg/mL were 60% (neat), 65% (two-fold dilution) and 74% (four-fold dilution), respectively, when compared with identical levels of p3-Alc_α_35 that were present in buffer A (closed diamonds).

This result confirmed that both adult serum and plasma contained factor(s) that interfered with the determination of p3-Alc_α _by ELISA even following a four-fold dilution (Figure 2A). This suggested to us that chemical separation of p3-Alc with quantitative recovery would be necessary to remove the interfering factor(s), and therefore to optimize the assay of p3-Alc in plasma or serum. A standard organic extraction protocol (chloroform:methanol 2:1) was used to recover synthetic p3-Alc_α_35 peptide from neat samples of healthy human serum (Figure 2B, **left**, squares) and plasma (Figure 2B, **right**, squares). After using this extraction protocol to pre-process samples, we observed a standard curve whose slope was almost identical to that of the standard curve in which identical amounts of the synthetic p3-Alc_α_35 peptide were dissolved in buffer A (closed diamonds). This indicated that factor(s) interfering with the immunoreaction were largely excluded by the chloroform:methanol extraction (see *Materials and Methods*). This procedure resulted in yields (according to ELISA) that were 82% for peptide (625 pg/mL) dissolved in serum (left) and 91% for that dissolved in plasma (right) (Figure 2B), relative to the identical concentration of peptide dissolved in buffer A. Recovery of p3-Alc_α_35 from plasma via the extraction protocol was high enough (> 90%) to be considered quantitative.

Once extraction conditions were established, we turned to the measurement of endogenous p3-Alc_α _levels in serum and plasma from various clinical populations. Serum and plasma samples derived from the same subjects (healthy normal, n = 5) were compared for p3-Alc_α _levels (Table 1). The p3-Alc_α _levels in serum samples were lower (67-87%) than those observed in plasma samples, suggesting that 10 to 30% of p3-Alc_α _may be trapped in clots or degraded by the proteases involving in clotting. Similar issues have also plagued Aβ ELISAs, and we therefore used plasma samples for analyzing the cohorts of subjects with various neurological diagnoses.

**Table 1:** p3-Alc_α _levels in serum and plasma derived from the same subjects.

	p3-Alcα (pg/mL)	
	
	Serum	Plasma
	
	Mean ± SD	Mean ± SD
1	129 ± 3	148 ± 6

2	103 ± 8	154 ± 15

3	142 ± 2	165 ± 0

4	151 ± 24	178 ± 13

5	182 ± 12	245 ± 10

Serum and plasma samples were prepared from five non-AD healthy volunteers respectively and p3-Alc_α _was quantified with ELISA in duplicates after extraction, as described in "*Materials and Methods*". SD, standard deviation.

### Plasma p3-Alc_α _levels in AD and FTLD subjects (Japanese cohort 1)

**Figure 3:**
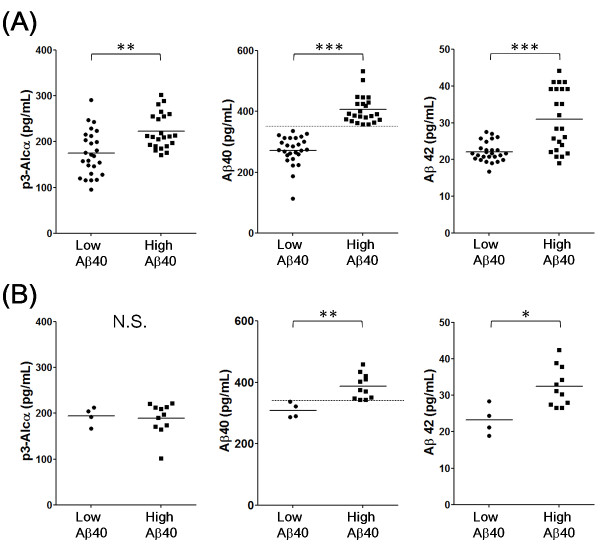
**Levels of p3-Alc_α _and Aβ in plasma from "low Aβ40" and "high Aβ40" populations of AD and FTLD subjects (Japanese cohort 1)**. Subjects with AD and FTLD were separated into two populations, who showed low (< 340 pg/mL) and high (> 340 pg/ml) Aβ40 levels (cut-off line is shown in the middle panels with dotted line). AD (**A**) and FTLD (**B**) are respectively indicated in two populations for p3-Alc_α _(left), Aβ40 (middle) and Aβ42 (right). Statistical analysis was performed using the Mann-Whitney U-test. *, *P *< 0.05; **, *P *< 0.01; ***, *P *< 0.001. N.S, not significant.

As a first trial, we examined levels of p3-Alc_α_, Aβ40, and Aβ42 levels in plasma from a cohort of patients (designated "Japanese cohort 1") with either AD (n = 49) or FTLD (n = 15). There were no remarkable differences for p3-Alc_α_, Aβ40, and Aβ42 levels between AD and FTLD subjects, except that there was a trend toward higher plasma Aβ40 values in some subjects (Table 2**and **Additional file 1, **Figure S2**). In order to characterize the "high Aβ40 subjects" and "low Aβ40 subjects" in further detail, a cut-off value of Aβ40 (340 pg/mL, the average of Aβ40 value in AD subjects), was imposed, and the p3-Alc_α_, Aβ40 and Aβ42 levels were re-analyzed (Figure 3). When populations were stratified by Aβ40 levels ("low Aβ40" indicates < 340 pg/mL; "high Aβ40" indicates > 340 pg/mL of middle panels of Figure 3), the high Aβ40 population was noted to contain samples with a significantly higher Aβ42 levels when compared to the Aβ42 levels from the low Aβ40 population, and this was true in both AD (right of Figure 3A) and FTLD (right of Figure 3B) populations. There were no significant differences between the low and high Aβ40 populations with respect to age, Aβ40/42 ratio, and MMSE score distributions (Additional file 1, **Figure S3**). However, the p3-Alc_α _levels were significantly higher in high Aβ40 samples when compared with those present in low Aβ40 samples (left of Figure 3A), indicating that levels of p3-Alc_α_, Aβ40 and Aβ42 were correlated in some subgroups. This coordinated alteration in p3-Alc_α _levels was observed in AD but not in FTLD subjects despite of the covariance of Aβ42 and Aβ40 levels in both populations (left of Figure 3B).

**Table 2:** Summary of subject information (Japanese cohort 1)

		N	Age	p3-Alcα (pg/mL)	Aβ 40 (pg/mL)	Aβ 42 (pg/mL)
AD	Total	49	75 ± 7	197 ± 50	335 ± 82	26 ± 7
	
	Male	18	76 ± 6	193 ± 57	342 ± 77	28 ± 8
	
	Female	31	75 ± 7	200 ± 47	331 ± 86	25 ± 7

FTLD	Total	15	64 ± 12	190 ± 31	366 ± 51	30 ± 6
	
	Male	8	65 ± 14	197 ± 20	371 ± 57	29 ± 7
	
	Female	7	61 ± 9	183 ± 41	361 ± 47	31 ± 7

Average age, gender, and average values of p3-Alc_α_, Aβ40 and Aβ42 of AD and FTLD subjects are summarized. Numbers indicate means ± standard deviation. Details of individual subjects are shown in Additional file 2, Tables S1 (AD) and S2 (FTLD).

Despite the fact that FTLD serves as a useful "other neurological disease" (OND) control, Japanese cohort 1 does not include non-demented subjects, and FTLD subjects do not match AD subjects with regard to average age (Table 2), therefore, we next examined plasma p3-Alc_α _levels in Japanese cohort 2 which included age-matched nondemented controls.

### Plasma p3-Alc_α _levels in AD and non-AD subjects (Japanese cohort 2)

**Figure 4:**
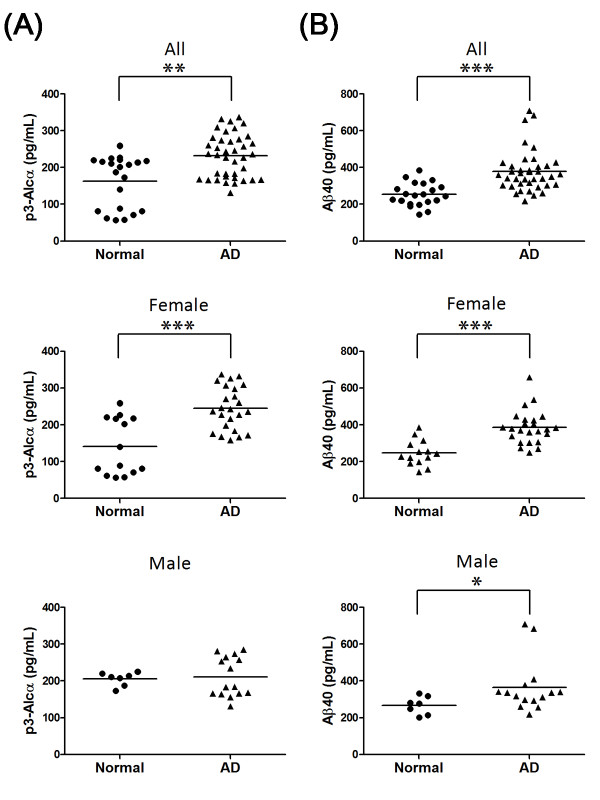
**Levels of p3-Alc_α _and Aβ in plasma from clinical populations (Japanese cohort 2)**. Plasma samples from AD subjects (n = 39) and age-matched nondemented controls (n = 21) were analyzed for levels of p3-Alc_α _**(A) **and Aβ40 **(B)**. Total subjects (All, upper panels), female subjects (Female, middle panels) and male subjects (Male, lower panels) are respectively indicated. Statistical analysis was performed using the Mann-Whitney U-test. **, *P *= 0.0017 (A, upper). ***, *P *= 0.0006 (A, middle). ***, *P *< 0.0001 (B, upper). ***, *P *< 0.0001 (B, middle). *, *P *= 0.0263 (B, lower).

We examined p3-Alc_α _levels in the extracted plasma of a second group of AD subjects and age-matched elderly nondemented controls (total n = 60). This cohort included AD patients of CDR 2 and CDR 3, and these AD patients showed relatively higher Aβ40 levels (n = 39, 378 ± 113 pg/mL) in plasma when compared to those from the age-matched normal control subjects (n = 21, 254 ± 63 pg/mL) (Table 3). The plasma of these AD subjects (n = 39, average age 76 ± 7) contained 232 ± 59 pg/mL p3-Alc_α_, while that of age-matched normal controls (n = 21, average age 73 ± 6) contained 163 ± 70 pg/mL of p3-Alc_α _(Table 3, Figure 4A, **upper; ***P *= 0.0017 by the Mann-Whitney U-test)). Surprisingly, the statistical difference was largely restricted to female subjects. The plasma from female AD subjects (n = 24) contained 245 ± 58 pg/mL of p3-Alc_α_, while that of female elderly nondemented controls (n = 14) contained 142 ± 77 pg/mL (*P *= 0.0006) (Figure 4A, **middle**). In contrast, plasma of male AD subjects (212 ± 54 pg/mL, n = 15) and male elderly normal controls (205 ± 19 pg/mL, n = 7) showed similar levels of p3-Alc_α _and no statistical significance was detected (Figure 4A, **lower**).

**Table 3:** Summary of subject information (Japanese cohort 2)

		N	Age	p3-Alcα (pg/mL)	Aβ 40 (pg/mL)
AD	Total	39	76 ± 7	232 ± 59	378 ± 113
	
	Male	15	74 ± 7	212 ± 54	366 ± 143
	
	Female	24	78 ± 7	245 ± 58	386 ± 92

Normal	Total	21	73 ± 6	163 ± 70	254 ± 63
	
	Male	7	71 ± 7	205 ± 19	267 ± 49
	
	Female	14	75 ± 6	142 ± 77	247 ± 69

Average age, gender, and average values of p3-Alc_α _and Aβ40 are summarized. Numbers indicate means ± standard deviation. Details of individual subjects are shown in Additional file 2, Tables S3 (AD) and S4 (Normal).

Unextracted plasma samples from this cohort were also studied. Aβ40 levels in AD subjects distributed within a higher range than that observed for age-matched normal controls (Figure 4B, **upper**). The higher plasma Aβ40 levels in female, but not male, AD subjects were readily obvious to visual inspection (Figure 4B, **middle, lower**). Levels of p3-Alc_α _and Aβ40 were increased in parallel in AD subjects and were higher than the levels observed in elderly normal controls.

**Figure 5:**
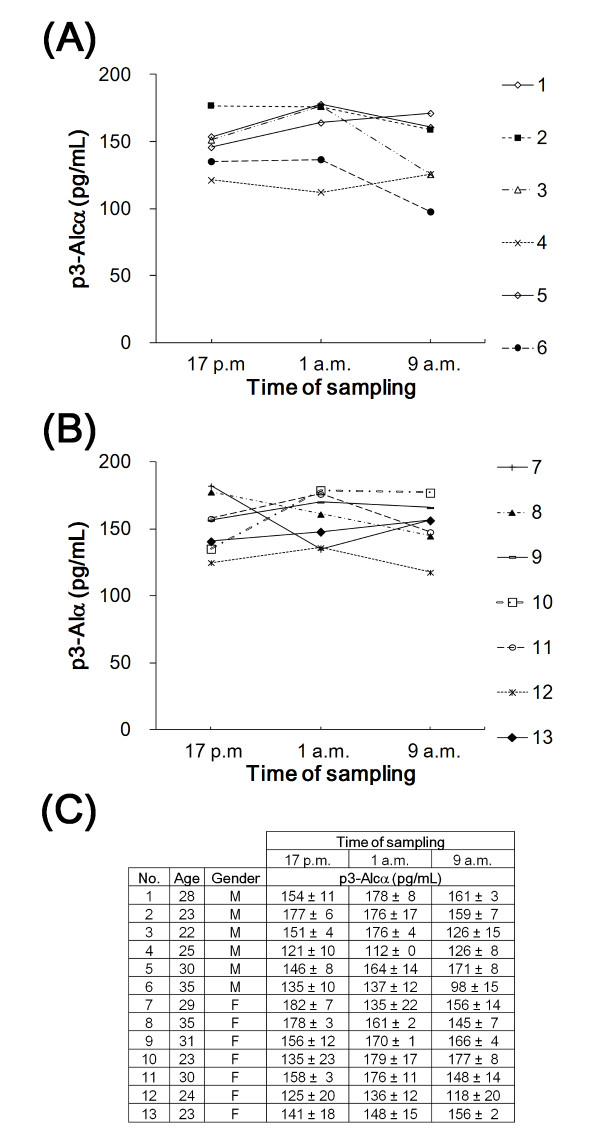
**Diurnal changes of serum p3-Alc_α _levels over a 24 hr period**. Sera of young healthy volunteers (**A**, 6 males; **B**, 7 females) were examined for p3-Alc_α _contents every 8 h within a day. Sampling times (5 p.m., 1 a.m. and 9 a.m.) are indicated. Clinical information about the volunteers and values for p3-Alc_α _levels in sera are described in panel **C**.

The p3-Alc_α _levels within a particular individual did not substantially change when blood samples were taken every 8 h over a 24 h period (Figure 5). Therefore, the possibility that the time of day when blood was sampled was excluded as a significant factor.

### Correlation between levels of p3-Alc_α _and Aβ40

**Figure 6:**
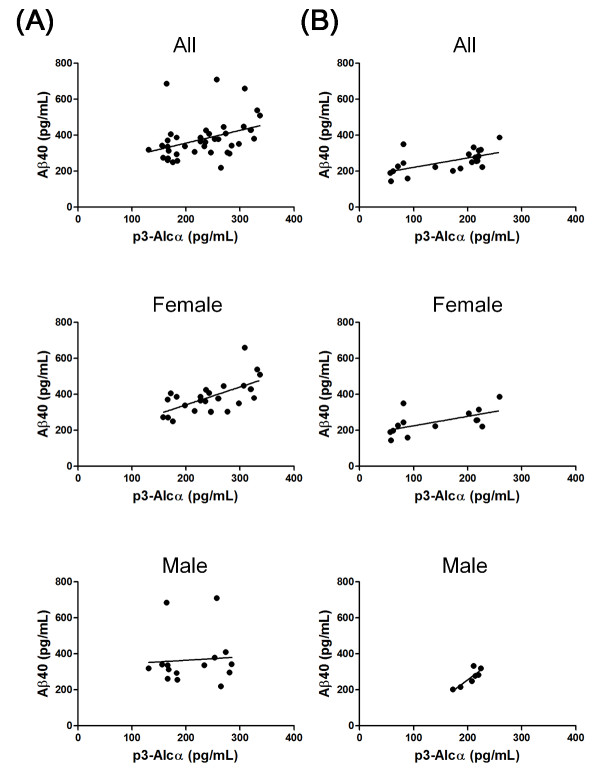
**Correlation between p3-Alc_α _and Aβ40 levels in AD and normal subjects (Japanese cohort 2)**. Correlation of plasma p3-Alc_α _and Aβ levels was explored in AD subjects (**A**) and normal controls (**B**). Total subjects (All, upper panels), female subjects (Female, middle panels) and male subjects (Male, lower panels) are respectively indicated. Statistical analysis was performed by using the Pearson's correlation coefficient. A. Upper; r = 0.4405 (*P *= 0.0050), Middle; r = 0.6149 (*P *= 0.0014), Lower; r = 0.07328 (*P *= 0.7952). B. Upper; r = 0.6275 (*P *= 0.0023), Middle; r = 0.6117 (*P *= 0.0201), Lower; r = 0.7857 (*P *= 0.0480).

We anticipated that the level of non-aggregatable p3-Alc_α _in plasma might parallel that of Aβ, at least with regard to Aβ40, which is less aggregatable than Aβ42. Therefore, we tested for the existence of any correlation between levels of p3-Alc_α _and those of Aβ40 in Japanese cohort 2. In AD subjects (n = 39), a significant correlation was observed between levels of p3-Alc_α _and Aβ (Figure 6A upper; r = 0.4405, *P *= 0.0050). However, this strong correlation was restricted to samples from female subjects (n = 24) (Figure 6A middle; r = 0.6149, *P *= 0.0014). No significant correlation was observed between levels of p3-Alc_α _and Aβ in samples from male subjects (n = 15) (Figure 6A, lower; r = 0.07328, *P *= 0.7952).

Analysis of levels of p3-Alc_α _and Aβ in samples from normal subjects revealed that there was also a strong correlation in normal subjects (n = 21) between levels of these two peptides (Figure 6B, upper; r = 0.6275, *P *= 0.0023). The correlation between levels of p3-Alc_α _and Aβ in samples from both normal females (n = 14) and those from normal males (n = 7) was statistically significant (for female subjects, Figure 6B, middle; r = 0.6117, *P *= 0.0201: for male subjects, Figure 6B, lower; r = 0.7857, *P *= 0.0480). Overall, these analyses indicated that there was a significant correlation between levels of p3-Alc_α _and those of Aβ40 in the plasma of normal subjects, and that this correlation was more robust in the plasma of female AD subjects.

## Discussion

The pathogenesis of sporadic AD is thought to be genetically heterogeneous. The precise identities and relative importance of the many contributory genes remain unclear, and additional loci have recently been identified [21]. This is in contrast to the situation for FAD in which pathogenic mutations of APP and PS genes cause alterations in Aβ speciation [6,7].

From the analyses of p3-Alc_α _levels in CSF, we previously proposed that alternative processing of multiple γ-secretase substrates may occurs in some sporadic AD populations. This earlier study was performed using a combination of immunoprecipitation and MALDI-TOF/MS to isolate and quantify p3-Alc in 300 μL of CSF [4,5]. Although the procedure showed semi-quantitative accuracy and was effective for testing changes in the abundance of p3-Alc species in the brain, CSF sampling is relatively invasive (when compared with blood sampling) and presents a challenge for large and/or longitudinal studies that involve repeated sampling over time. Therefore, we developed a system by which p3-Alc could be quantified in relatively small plasma samples. The combination of simple p3-Alc extraction and highly selective ELISA enabled the quantification of p3-Alc_α _in 100 μL of plasma (200 μL in duplicate assay). Successful extraction of p3-Alc_α _from plasma with chloroform/methanol solvent suggests that the majority of p3-Alc_α _in plasma may be bound to lipid or lipoproteins. While we have not performed further characterization of p3-Alc_α _in plasma, apolipoprotein E would be a potential suspect and association could vary by *APOE *isotype. A study of the effect of this variable in both CSF and plasma samples is underway.

We have not yet developed an ELISA system that can perfectly discriminate levels of each individual p3-Alc_α _species (e.g., p3-Alc_α_35, p3-Alc_α_38). Such an assay requires antibodies against the neoepitopes created when the specific C-termini are generated by γ-cleavage. Since the polyclonal 839 antibody was prepared using the C-terminal amino acid sequence of p3-Alc_α_35 as antigen, the ELISA used in this study displayed a relative specificity for detecting synthetic p3-Alc_α_35, but there was also crossreactive detection of p3-Alc_α_39 synthetic peptide at about 50-60% of the sensitivity that was observed for detection of p3-Alc_α_35 (Figure 1B).

p3-Alc_α_35 with a C-terminal Thr851 is the major (~90%) peptide among the p3-Alc_α _species with various C-termini [4,5]. Thus, we conclude that the increased level of p3-Alc_α _in AD subjects is probably largely dependent on an increase of p3-Alc_α_35, although we cannot yet exclude the possibility that another species such as p3-Alc_α_38 may be selectively increased in AD subjects [5]. Therefore, in the present study, we could not examine *qualitative *changes of p3-Alc_α _species in plasma. However, we have successfully developed a potentially useful ELISA for p3-Alc peptides and demonstrated its utility in the detection and quantification of plasma p3-Alc_α _peptide. Despite the fact that plasma levels of p3-Alc_α _are about ten-fold less than the levels in CSF, p3-Alc_α _levels are readily quantifiable in both body fluids using this novel ELISA (**Hata *et al.***, in preparation).

Using the novel ELISA specific for p3-Alc_α_, we found that plasma p3-Alc_α _levels were significantly higher in some AD subjects who also showed higher Aβ40 levels. In female subjects, an obvious and statistically significant difference in plasma p3-Alc_α _levels was evident when AD subjects were compared against age-matched elderly nondemented controls. The explanation for why p3-Alc_α _levels and Aβ40 levels are higher in some female AD subjects remains unclear. It is worth noting that gonadal hormones are known to regulate generation of Aβ [22-24] as well as transcription of the *APOE *gene [25]. Similar gender dimorphic results were recently obtained in plasma samples of another independent cohort when the same ELISA system was employed, suggesting that selection bias is probably not the explanation for the sex-related differences (**Kamogawa *et al.***, in preparation).

Increased levels of plasma Aβ and p3-Alc_α _in some AD subjects may suggest a γ-secretase enzymopathy, as proposed in analyses of CSF p3-Alc [5] and CSF APLP1-28 [17]. However, we cannot exclude the possibility that p3-Alc_α _clearance may be decreased in AD, especially since evidence for defective Aβ clearance has been presented by other investigators [18,26]. Analyses of the possible roles for *APOE *genotype, CSF and plasma apoE levels, and CSF and plasma γ-secretase and p3-Alc_α_-degrading enzyme activities are all underway. It is worth noting that polymorphisms in *APOE *[27] and neprilysin [28] have been linked to increased risk of chronic traumatic encephalopathy and/or AD following traumatic brain injury [29], and these identical factors could play similar roles here.

In Japanese cohort 2, we observed higher levels of plasma Aβ40 and p3-Alc_α_. Since p3-Alc_α _peptides are not deposited in brain or cerebral vessels, the molecular pathogenesis underlying these changes in CSF and plasma p3-Alc levels remains unknown. Serial studies of both plasma and CSF from the same individuals over time are required in order to elucidate the underpinnings of these changes, including the sexual dimorphism. Serial studies of CSF and plasma p3-Alc_α _from transgenic animals and from subjects in the National Institute on Aging Alzheimer's Disease Neuroimaging Initiative should help clarify these results, and those studies are also underway.

The p3-Alc_α _in blood is likely to be derived from largely brain because Alc_α _expresses predominantly in brain neurons [1] and transgenic mice expressing human Alc_α_-CTF driven by a neuron-specific PDGF promoter increased p3-Alc_α _levels in plasma (Hata and Takei, unpublished observation). Alcadein is almost identical to APP with respect to neural expression, cellular distribution, metabolism, and function [1-4]. Furthermore, we would re-emphasize that p3-Alc does not aggregate and is stable in plasma, both of which are desirable qualities in clinical sample preparation and handling and both of which have complicated analysis of Aβ in bodily fluids. These properties are useful for the development of sensitive procedures for the detection of γ-secretase dysfunction, and p3-Alc_α _plasma levels may be useful in identifying AD subjects whose clinical phenotype is caused by a functional alteration of γ-secretases and/or by defective clearance of transmembrane domain-derived peptides. This endophenotyping may be important for selecting subjects for trials of γ-secretase modulators or plasminogen activator inhibitor-1 inhibitors [30,31], respectively.

## Materials and methods

### Blood collection and processing, and quantification of plasma p3-Alc_α_

Informed consent for the use of all human plasma and serum in this study was obtained from the patients and/or their families and approved by the appropriate ethical boards at each institution. Alzheimer's disease was clinically diagnosed based on two major criteria; Diagnostic and Statistical Manual of Mental Disorders: 4th Edition (DSM-IV) and National Institute of Neurological and Communicational Disorders and Stroke - Alzheimer's Disease and Related Disorders Association (NINCDS-ADRDA). In Japanese cohort 1, the clinical diagnoses of patients with FTLD were made on the basis of established clinical criteria [32]. A compilation of clinical characteristics and data is shown in Table 2. Detailed descriptions of all subjects are shown in Additional files 2, **Tables S1 and S2**.

In Japanese cohort 2, all subjects with AD are inpatients and showed MMSE scores consistent with a diagnosis of either moderate or severe dementia (CDR 2 or CDR 3). Clinical characteristics and data are shown in Table 3. Detailed descriptions of all subjects including the raw values of p3-Alc_α _are shown in Additional files 2, **Tables S3 and S4**.

For plasma samples, blood was collected into tubes containing EDTA and centrifuged. For serum samples, blood was collected into tubes with no EDTA and allowed to coagulate. To extract p3-Alc_α _peptides, a four-fold volume of organic reagent (800 μL of chloroform: methanol [2:1] mixture) was added to plasma or serum (usually 200 μL for duplicate assay) in a conical tube (1.5 mL), agitated well by vortexing or sonication for 10 s, and then left to stand for 1 h at room temperature (18-23°C). Next, a 160 μL aliquot of distilled water was added, and the samples were mixed by vortexing. The samples were centrifuged at 15,000 rpm for 15 min, and the aqueous phase was recovered and dried using a SpeedVac system. The dried sample was dissolved in 250 μL of PBS containing 1% (w/v) BSA and 0.05% (v/v) Tween-20 (buffer A). The samples (×1) or samples further diluted with buffer A (2- and 4-fold) were used for ELISA. Aliquots of 100 μL were studied in duplicate. Generally the undiluted sample (×1) was examined for quantification.

### Antibodies and ELISA system

The major p3-Alc_α _species, p3-Alc_α_35, is a peptide that includes the sequence from Ala^817 ^to Thr^852 ^of Alc_α_1 (for the amino acid sequence of p3-Alc_α_, see Additional file 1, **Figure S1A)**. Polyclonal rabbit antibodies were raised against a peptide containing the sequence between position 817 and 822 (#817 antibody) and a peptide containing the sequence between position 839 and 852 (#839 antibody), and affinity purified with the each respective peptide antigen resin. The antibody 839 was used to capture p3-Alc_α_, and horseradish peroxidase-conjugated pan p3-Alc_α _antibody 817 and tetramethyl benzidine were used to detect the captured p3-Alc_α _colormetrically at OD_450_. Synthetic p3-Alc_α_35 peptide was used as a standard assay protein. The specificity for p3-Alc_α _in the ELISA was confirmed using synthetic p3-Alc_α_35 (major species of CSF p3-Alc_α_), p3-Alc_α_39 and p3-Alc_β_37 peptides [4,5]. Aβ in neat samples without extraction was quantified with sELISA (Wako Pure Chemical Industries Co Ltd for cohort 1 and IBL Co Ltd for cohort 2).

## Abbreviations

AD: Alzheimer's disease; Aβ: amyloid β-protein; Alc: alcadein; APP: amyloid β-protein precursor; p3-Alc: small peptide generated by serial primary and secondary cleavages of Alc; CSF: cerebrospinal fluid: FAD: familial Alzheimer's disease; SAD: sporadic Alzheimer's disease; PS: presenilin; sELISA: sandwich enzyme-linked immunosorbent assay.

## Competing interests

The authors declare that they have no competing interests.

## Authors' contributions

TK and SH carried out all of the experiments. YH, YH-S and MM established and modified the p3-Alc_α _sELISA system. TY, TI and HA collected the blood samples for this study. TN, YS, TY, HA, SG and TS participated in the design of the study, and TS conceived the study. SG, HA and TS are co-senior authors. All authors read and approved the final manuscript.

## Supplementary Material

Additional file 1 **Figure S1. Amino acid sequences of p3-Alc_α_, and recovery of p3-Alc_α _in sera by immunoprecipitation and detection of p3-Alc_α _by Western blotting**. A. Amino acid sequences and cleavage sites of p3-Alc_α _and Aβ in human. The amino acid sequences of p3-Alc_α _species and the primary α- and secondary γ-cleavage sites of Alcα_1 _are indicated. "35" indicates major γ-cleavage site to generate p3-Alc_α_35 while "38" indicates minor γ-cleavage site to generate p3-Alc_α_38. The amino acid sequences of p3 and Aβ peptides are also shown. Primary α- and β- cleavage sites of APP695 are shown, "40" indicates major γ-cleavage site to generate Aβ40 while "42" indicates minor γ-cleavage site to generate Aβ42. Putative transmembrane region is indicated with box. B. Detection of endogenous and synthetic p3-Alc_α _peptides. Synthetic p3-Alc_α_35 peptide (10 ng) was added to human serum (HS), calf serum (CS), fetal bovine serum (FBS), and bovine serum albumin solution in PBS (BSA; 10 mg/mL). The samples with (+) or without (-) addition of p3-Alc_α_35 peptide were subject to immunoprecipitation with anti-pan p3-Alc_α _3B5 antibody and immunoprecipitates were analyzed by Western blotting with UT135 antibody. The far right lane is a loading control sample containing 4 ng of p3-Alc_α_35 peptide. "*Overexposure*" (lower row) indicates overexposure of film. The pan p3-Alc_α _mouse monoclonal antibody 3B5 and polyclonal rabbit antibody UT135 have been described (*J. Biol. Chem*. [2009] 284, 36024-36033). **Figure S2. Levels of p3-Alc_α _in plasma of AD and FTLD subjects (Japanese cohort 1)**. Plasma samples from AD (n = 49) and FTLD (n = 15) subjects were analyzed for levels of p3-Alc_α_. Statistical analysis was performed using the Mann-Whitney U-test. N.S, not significant. **Figure S3. Age, Aβ40/42 ratio and MMSE score distribution in subjects of low and high Aβ40 populations (Japanese cohort 1)**. Age (upper left), Aβ40/42 ratio (upper right) and MMSE score (lower left) of AD and FTLD subjects of low Aβ40 population are compared to these of high Aβ40 population. Statistical analysis was performed using the Mann-Whitney U-test. N.S, not significant. Click here for file

Additional file 2 **Table S1. Information on AD subjects (Japanese cohort 1)**. The subjects (n = 49) were clinically diagnosed using CDR (clinical dementia rating) criteria. **Table S2. Information on FTLD subjects (Japanese cohort 1)**. The patients (n = 15) were clinically diagnosed as described in *"Materials and Methods"*. **Table S3. Information on AD subjects (Japanese cohort 2)**. The subjects (n = 39) were clinically diagnosed with AD at stages CDR 2 or CDR 3. **Table S4. Information of normal controls (Japanese cohort 2)**. Age-matched normal elderly controls (n = 21) are indicated. The subjects were clinically non-demented. Click here for file
