# Supplementary figures and images for: Coordinated increase of γ-secretase reaction products in the plasma of some female Japanese sporadic Alzheimer's disease patients: quantitative analysis of p3-Alcα with a new ELISA system
Source: Mol Neurodegener. 2011 Nov 8;6:76. doi: 10.1186/1750-1326-6-76 (PMC3247855; doi:10.1186/1750-1326-6-76)

(A)

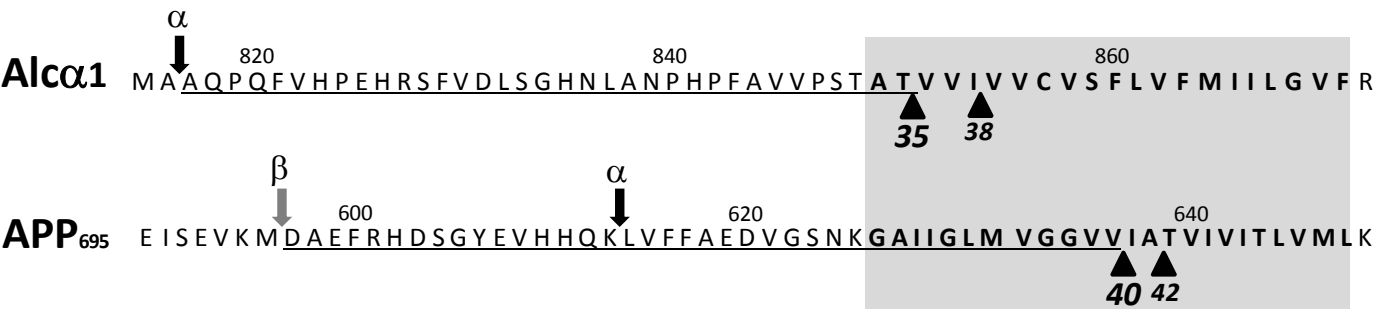

(B)

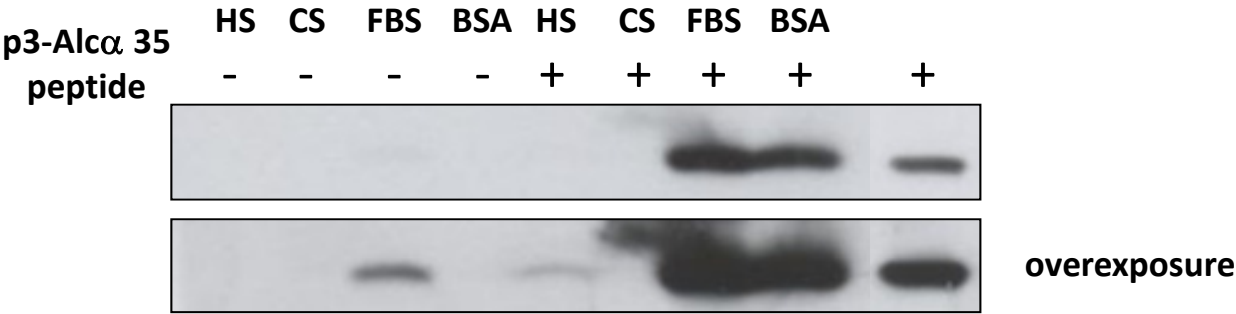

Fig. S1

# p3-Alcα (All)

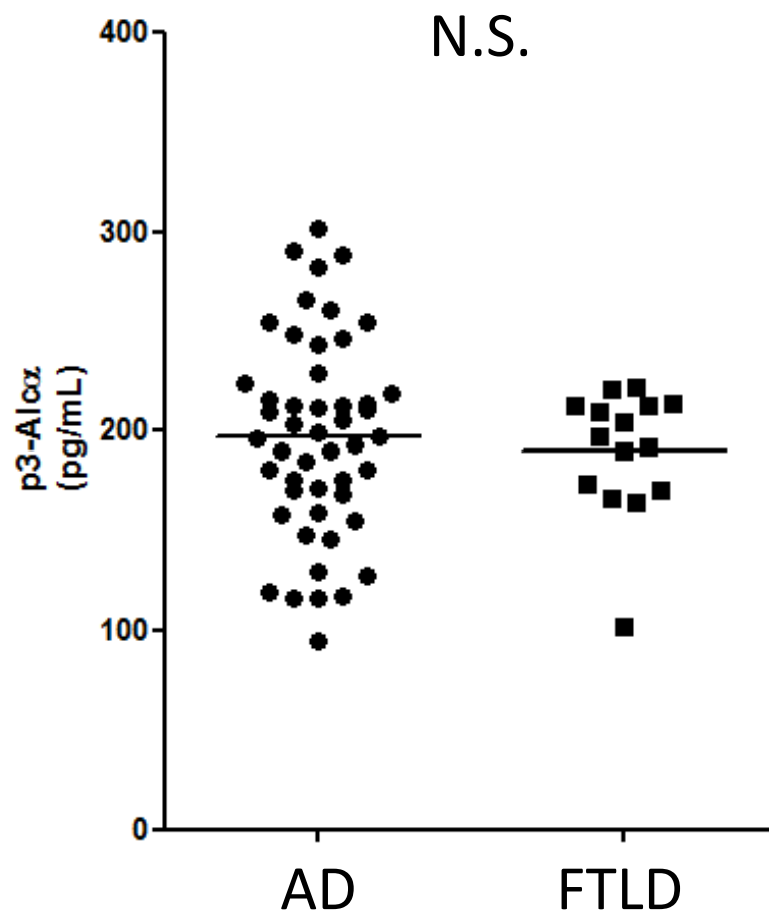

Fig. S2

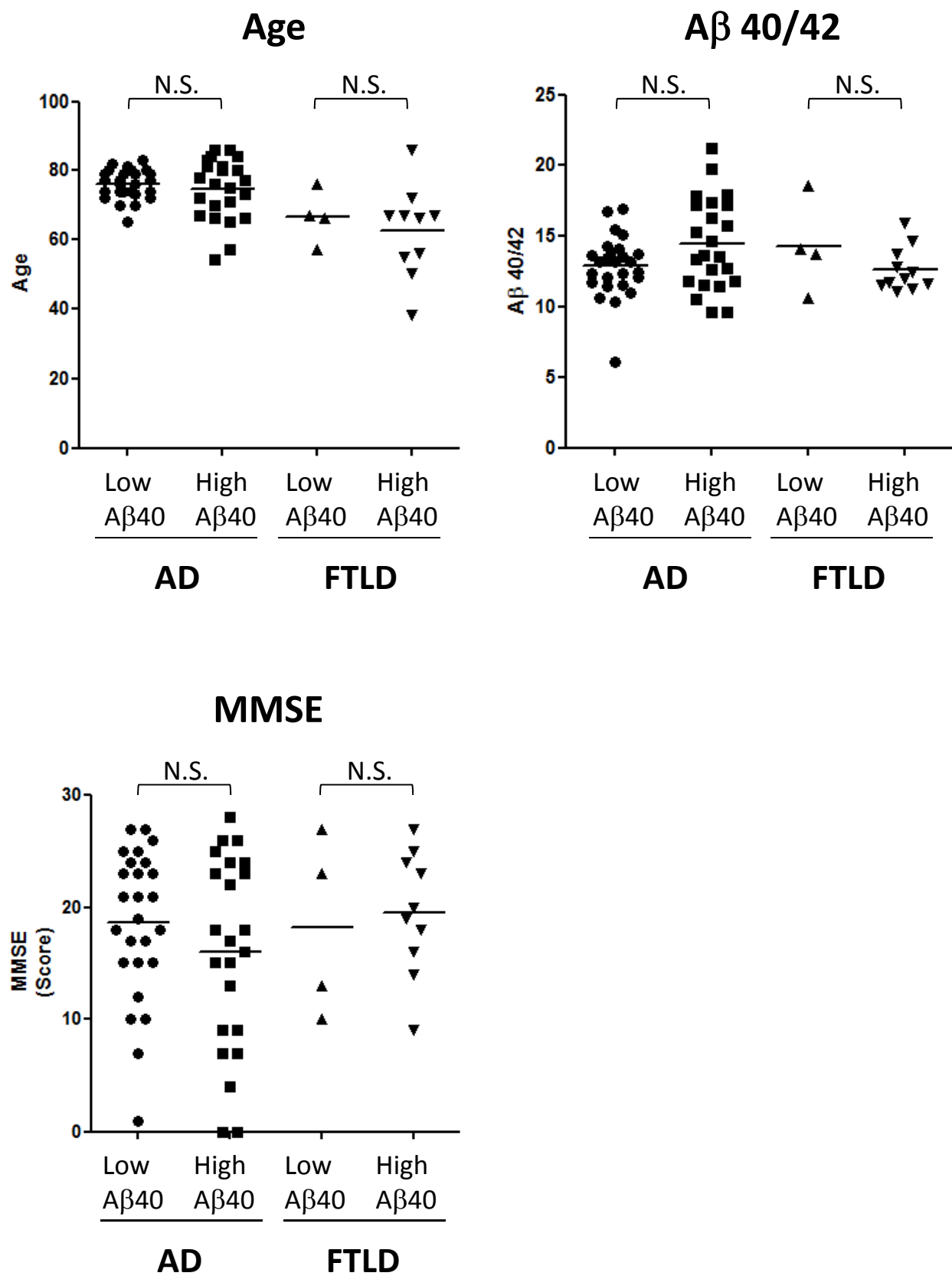

Fig. S3

Supplement: Additional file 1 — Figure S1. Amino acid sequences of p3-Alcα, and recovery of p3-Alcα in sera by immunoprecipitation and detection of p3-Alcα by Western blotting. A. Amino acid sequences and cleavage sites of p3-Alcα and Aβ in human. The amino acid sequences of p3-Alcα species and the primary α- and secondary γ-cleavage sites of Alcα1 are indicated. "35" indicates major γ-cleavage site to generate p3-Alcα35 while "38" indicates minor γ-cleavage site to generate p3-Alcα38. The amino acid sequences of p3 and Aβ peptides are also shown. Primary α- and β- cleavage sites of APP695 are shown, "40" indicates major γ-cleavage site to generate Aβ40 while "42" indicates minor γ-cleavage site to generate Aβ42. Putative transmembrane region is indicated with box. B. Detection of endogenous and synthetic p3-Alcα peptides. Synthetic p3-Alcα35 peptide (10 ng) was added to human serum (HS), calf serum (CS), fetal bovine serum (FBS), and bovine serum albumin solution in PBS (BSA; 10 mg/mL). The samples with (+) or without (-) addition of p3-Alcα35 peptide were subject to immunoprecipitation with anti-pan p3-Alcα 3B5 antibody and immunoprecipitates were analyzed by Western blotting with UT135 antibody. The far right lane is a loading control sample containing 4 ng of p3-Alcα35 peptide. "Overexposure" (lower row) indicates overexposure of film. The pan p3-Alcα mouse monoclonal antibody 3B5 and polyclonal rabbit antibody UT135 have been described (J. Biol. Chem. [2009] 284, 36024-36033). Figure S2. Levels of p3-Alcα in plasma of AD and FTLD subjects (Japanese cohort 1). Plasma samples from AD (n = 49) and FTLD (n = 15) subjects were analyzed for levels of p3-Alcα. Statistical analysis was performed using the Mann-Whitney U-test. N.S, not significant. Figure S3. Age, Aβ40/42 ratio and MMSE score distribution in subjects of low and high Aβ40 populations (Japanese cohort 1). Age (upper left), Aβ40/42 ratio (upper right) and MMSE score (lower left) of AD and FTLD subjects of low Aβ40 population are compared [file 1750-1326-6-76-S1.PDF]
