# Supplementary material for: Coordinated increase of γ-secretase reaction products in the plasma of some female Japanese sporadic Alzheimer's disease patients: quantitative analysis of p3-Alcα with a new ELISA system
Source: Mol Neurodegener. 2011 Nov 8;6:76. doi: 10.1186/1750-1326-6-76 (PMC3247855; doi:10.1186/1750-1326-6-76)
Supplement: Additional file 2 — Table S1. Information on AD subjects (Japanese cohort 1). The subjects (n = 49) were clinically diagnosed using CDR (clinical dementia rating) criteria. Table S2. Information on FTLD subjects (Japanese cohort 1). The patients (n = 15) were clinically diagnosed as described in "Materials and Methods". Table S3. Information on AD subjects (Japanese cohort 2). The subjects (n = 39) were clinically diagnosed with AD at stages CDR 2 or CDR 3. Table S4. Information of normal controls (Japanese cohort 2). Age-matched normal elderly controls (n = 21) are indicated. The subjects were clinically non-demented. [file 1750-1326-6-76-S2.PDF]

Japanese cohort 1

AD								
No.	CDR	Age	Gender	MMSE (Score)	p3-Alcα (pg/mL)	Aβ40 (pg/mL)	Aβ42 (pg/mL)	Clinilal diagnosis
1	3	70	M	1	155	243	22.1	AD
2	1	80	F	23	116	222	21.7	AD
3	2	80	F	15	213	274	27.5	AD
4	1	86	M	16	214	446	39.3	AD
5	1	79	F	23	148	313	24.8	AD
6	2	82	M	19	128	312	26.2	AD
7	2	74	F	15	95	260	19.0	AD
8	1	77	M	21	169	288	19.4	AD
9	3	78	M	4	219	430	39.3	AD
10	2	83	M	17	247	272	20.8	AD
11	1	74	F	10	196	285	25.7	AD
12	1	76	F	22	266	532	41.1	AD
13	1	72	M	26	190	357	20.8	AD
14	0.5	72	F	23	130	265	20.3	AD
15	2	79	M	7	116	291	23.0	AD
16	1	71	M	18	210	503	41.1	AD
17	1	77	F	17	159	240	20.8	AD
18	1	77	F	25	158	224	21.2	AD
19	1	76	M	27	146	257	21.7	AD
20	1	70	F	27	181	326	25.7	AD
21	1	81	F	25	211	418	32.0	AD
22	2	77	F	9	181	363	28.4	AD
23	2	79	M	10	120	274	21.2	AD
24	1	79	F	25	176	318	19.9	AD
25	1	73	F	24	212	369	22.1	AD
26	2	57	F	7	171	358	21.7	AD
27	2	70	F	15	261	446	39.3	AD
28	3	66	F	0	213	374	24.8	AD
29	1	54	F	23	190	383	26.2	AD
30	2	80	F	0	185	386	19.0	AD
31	1	74	F	18	224	113	19.4	AD
32	1	67	M	28	193	362	39.3	AD
33	1	65	F	21	216	336	20.8	AD
34	2	84	M	13	255	425	25.7	AD
35	2	65	M	9	206	380	41.1	AD
36	1	66	F	17	176	393	20.8	AD
37	1	80	F	24	243	297	22.6	AD
38	1	74	M	26	199	322	27.1	AD
39	1	73	F	21	203	262	22.6	AD
40	1	75	M	18	302	425	35.2	AD
41	2	74	M	12	291	268	19.9	AD
42	2	86	F	7	289	400	28.4	AD
43	1	80	F	23	282	391	35.2	AD
44	2	81	F	15	249	448	44.2	AD
45	1	72	F	15	229	187	16.7	AD
46	1	84	F	24	197	373	23.9	AD
47	1	81	M	18	117	301	22.6	AD
48	1	76	F	24	172	313	21.7	AD
49	1	83	F	26	255	386	22.6	AD

Table S1

Japanese
cohort 1

FTLD							
No.	Age	Gender	MMSE (Score)	p3-Alcα (pg/mL)	Aβ40 (pg/mL)	Aβ42 (pg/mL)	Clinilal diagnosis
1	67	M	18	221	421	27.5	FTLD
2	67	M	23	174	351	26.6	FTLD
3	66	F	27	167	289	28.4	FTLD
4	50	F	16	102	402	37.9	FTLD
5	66	M	25	213	410	34.3	FTLD
6	69	F	19	210	347	30.2	FTLD
7	67	M	13	213	287	21.2	FTLD
8	55	F	14	171	343	28.0	FTLD
9	67	F	9	214	434	38.8	FTLD
10	56	M	24	197	375	26.6	FTLD
11	86	M	19	165	459	42.4	FTLD
12	38	M	27	190	343	31.1	FTLD
13	72	F	20	222	371	33.0	FTLD
14	57	F	10	192	337	19.0	FTLD
15	76	M	23	205	321	24.4	FTLD

Table S2

Japanese
cohort 2

AD							
No.	CDR	Age	Gender	MMSE (score)	p3-Alcα (pg/mL)	Aβ40 (pg/mL)	Clinical diagnosis
1	3	84	F	0	277	303	AD
2	3	76	F	0	198	338	AD
3	3	78	F	0	337	508	AD
4	3	77	M	10	253	378	AD
5	3	70	M	0	156	340	AD
6	3	83	F	0	172	404	AD
7	3	82	F	0	309	658	AD
8	3	81	M	0	281	297	AD
9	3	84	F	0	243	406	AD
10	3	65	M	0	168	312	AD
11	3	79	F	10	158	273	AD
12	3	75	F	0	237	425	AD
13	3	74	F	0	166	370	AD
14	3	85	F	5	270	445	AD
15	3	75	M	0	184	256	AD
16	3	71	F	0	236	360	AD
17	3	79	F	0	320	428	AD
18	3	78	F	10	246	302	AD
19	3	65	M	0	285	341	AD
20	3	81	F	0	183	386	AD
21	3	74	M	0	131	318	AD
22	3	80	F	0	227	365	AD
23	3	78	F	0	326	379	AD
24	3	67	M	0	166	260	AD
25	3	80	M	0	166	336	AD
26	3	78	M	14	183	293	AD
27	3	64	F		167	270	AD
28	3	61	F		216	307	AD
29	3	80	F	2	298	350	AD
30	3	85	F		227	385	AD
31	3	61	F		260	377	AD
32	3	80	F	0	176	248	AD
33	3	74	M	0	234	337	AD
34	3	80	F	0	307	447	AD
35	3	85	M	0	257	708	AD
36	3	70	M	0	164	685	AD
37	2	84	M	20	274	409	AD
38	2	83	F	17	332	537	AD
39	2	64	M	25	265	218	AD

Table S3

Japanese
cohort 2

Normal							
No.	CDR	Age	Gender	MMSE (score)	p3-Alcα (pg/mL)	Aβ40 (pg/mL)	Clinical diagnosis
1	0	66	M		208	248	Normal
2	0	77	F		62	198	Normal
3	0	66	F		58	144	Normal
4	0	71	F		71	226	Normal
5	0	80	F		89	158	Normal
6	0	72	F		81	348	Normal
7	0	71	F		216	254	Normal
8	0	65	F		57	190	Normal
9	0	70	M		214	277	Normal
10	0	81	F		227	221	Normal
11	0	69	M		225	318	Normal
12	0	65	M		220	282	Normal
13	0	68	F		202	293	Normal
14	0	75	F		218	256	Normal
15	0	79	F		221	314	Normal
16	0	79	F		81	244	Normal
17	0	78	F		259	386	Normal
18	0	78	M		173	201	Normal
19	0	64	M		187	214	Normal
20	0	82	F		140	222	Normal
21	0	83	M		211	332	Normal

Table S4
